# LPBF Manufactured Functionally Graded Lattice Structures Obtained by Graded Density and Hybrid Poisson’s Ratio

**DOI:** 10.3390/ma15124072

**Published:** 2022-06-08

**Authors:** Osama Abdelaal, Florian Hengsbach, Mirko Schaper, Kay-Peter Hoyer

**Affiliations:** 1 Department of Mechanical and Industrial Engineering, College of Engineering, Majmaah University, Al-Majmaah 11952, Saudi Arabia; 2 Mechanical Design and Production Engineering Department, Faculty of Engineering, Assiut University, Assiut 71516, Egypt; 3 Lehrstuhl für Werkstoffkunde (Materials Science), Paderborn University, Warburger Straße 100, 33098 Paderborn, Germany; hengsbach@lwk.upb.de (F.H.); schaper@lwk.upb.de (M.S.); hoyer@lwk.upb.de (K.-P.H.); 4 Direct Manufacturing Research Center (DMRC), Mersinweg 3, 33100 Paderborn, Germany

**Keywords:** laser powder bed fusion (LPBF), Ti-6Al-4V, auxetic metamaterials, hybrid Poisson’s ratio, functionally graded lattice structures, flexural testing, energy absorption

## Abstract

The additive manufacturing (AM) of innovative lattice structures with unique mechanical properties has received widespread attention due to the capability of AM processes to fabricate freeform and intricate structures. The most common way to characterize the additively manufactured lattice structures is via the uniaxial compression test. However, although there are many applications for which lattice structures are designed for bending (e.g., sandwich panels cores and some medical implants), limited attention has been paid toward investigating the flexural behavior of metallic AM lattice structures with tunable internal architectures. The purpose of this study was to experimentally investigate the flexural behavior of AM Ti-6Al-4V lattice structures with graded density and hybrid Poisson’s ratio (PR). Four configurations of lattice structure beams with positive, negative, hybrid PR, and a novel hybrid PR with graded density were manufactured via the laser powder bed fusion (LPBF) AM process and tested under four-point bending. The manufacturability, microstructure, micro-hardness, and flexural properties of the lattices were evaluated. During the bending tests, different failure mechanisms were observed, which were highly dependent on the type of lattice geometry. The best response in terms of absorbed energy was obtained for the functionally graded hybrid PR (FGHPR) structure. Both the FGHPR and hybrid PR (HPR) structured showed a 78.7% and 62.9% increase in the absorbed energy, respectively, compared to the positive PR (PPR) structure. This highlights the great potential for FGHPR lattices to be used in protective devices, load-bearing medical implants, and energy-absorbing applications.

## 1. Introduction

Lightweight metallic metamaterials are a promising and quickly developing class of structural materials and the demand for it is on the rise due to their superior strength-to-weight ratios. One of the most common types are lattice structures. Lattice structures are geometrically complex so the traditional manufacturing processes does not provide the successful manufacturing option. This necessitates the use of modern processing technologies.

The recent advancements in additive manufacturing (AM) technologies have opened up a great opportunity for the development of lattice structures with controllable properties and relative density gradients that will play an important role in the future of lightweight applications. As a new manufacturing technology that emerged in the late 1980s, additive manufacturing (3D printing) is gradually attracting interest by both academia and industry. By using AM processes, the part is built by adding material layer-by-layer directly from a computer-aided design (CAD) model. This additive building fashion enables the creation of intricate graded lattice structures and organic geometries that were previously difficult or impossible to fabricate using traditional manufacturing processes. Among the various AM techniques, laser powder bed fusion (LPBF) has been shown to be an attractive manufacturing route to the freeform fabrication of complex net-shape metal components with applications across a range of industrial sectors. In LPBF, objects are built by the selective melting and fusing of metallic powder layers with a focused high power density laser beam. LPBF is the ideal technique for metallic lattice manufacturing compared to other metal AM techniques such as directed energy deposition (DED) due to the high resolution and minimum layer thickness achieving a higher geometrical accuracy.

There is a growing interest in the potential offered by additively manufactured lattice structures for use in a range of medical, energy absorption, and optimized strength or stiffness applications. Therefore, there has been substantial progress in the AM and investigations of the mechanical behavior of metallic lattice structures.

So far, multiple types of uniform lattice structures have been fabricated via AM including body-centered cubic (BCC), BCC-Z, face-centered-cubic (FCC), FCC-Z, Octet, hexagonal, diamond, and cubic.

Among the metallic lattice structures, the gradient density lattice structure has attracted many research interests as a new class of cellular solid. The lattice structures will have a valuable combination of high porosity and high strength if the distribution of the pore size, pore shape, and relative density can be optimized [1]. Recent research efforts are directed toward additive manufacturing and the characterization of metallic functionally graded (FG) lattice structures. Al-Saedi et al. [2] studied the mechanical characteristics and energy absorption capability of the FG F2BCC-type made of an Al-12Si alloy fabricated by LPBF under compression loading. In another work, Ma et al. [3] studied the energy absorption characteristics of the FG Ti-6Al-4V lattice structure graded based on the minimal curved surface method then manufactured by LPBF, and tested it under quasi-static compression. The compression–compression fatigue behavior of the Ti-6Al-4V gyroid graded lattice structures fabricated by LPBF were investigated by Yang et al. [4]. Similarly, Chen et al. [5] assessed both the compression–compression fatigue behavior and biocompatibility of the FG tantalum produced by LPBF. Bai et al. [6] evaluated the quasi-static compression and fatigue behavior of LPBF Ti-6Al-4V, in addition to the influence of sandblasting and gradient direction on its mechanical response. Choy et al. [7] employed electron beam melting (EBM) to fabricate FG Ti-6Al-4V lattices. They reported that FG structures exhibited a progressive layer-by-layer deformation mode regardless of the unit cell designs and build direction. The compressive fatigue behavior of the LPBF-processed Ti-6Al-4V radially graded gyroids was studied by Mahmoud et al. [8]. The study focused on the manufacturability and influence of defects induced by LPBF on the uniform and FG gyroids. All studies reported promising results of the AM of metallic FG lattice structures in terms of the energy absorption capabilities compared to the uniform density counterparts.

Another subset of lattice structures that have received high interest within the AM research community during the last decade is the auxetic lattice structures that exhibit negative Poisson’s ratio (υ < 0) due to the novel behavior that they exhibit under deformation such as synclastic curvature, a general increase in the transverse shear modulus, acoustics attenuation, indentation, and flatwise compressive stiffness at low relative densities [9]. The reason behind this behavior is that auxetic materials and structures exhibit the very unusual deformation mechanism of becoming wider when stretched and narrower when compressed; that is, they have negative Poisson’s ratios (NPRs). Accordingly, due to this unique behavior, auxetic structures have been considered for a wide range of smart and functional devices including smart antennas, stretchable sensors, protective equipment, and in many other applications from the fields of nanotechnology and biomedicine to defense and aerospace. Despite these advantages, the complex designs of auxetic structures pose difficulties in manufacturing, and hence have limited their industrial applications [10]. Many theoretical studies have been conducted on auxetic lattice structures including topology optimization and design [11,12,13] and mechanical behavior evaluation [14,15,16]. On the other hand, there has recently been a considerable increase into the experimental investigations on the AM and characterization of metallic lattice structures with negative Poisson’s ratio driven by the improved capabilities of AM techniques. The influence of variation in the cubic chiral auxetic unit cell parameters on the compressive mechanical properties of the EBM-processed Ti-6Al-4V structure was investigated by Warmuth et al. [17]. Shen et al. [18] conducted a similar study on the compressive behavior of the Ti-6Al-4V 3D re-entrant lattice structure processed by EBM. Work by Novak et al. [19] also examined the mechanical behavior of auxetic cellular structures built by EBM from Ti-6Al-4V based on inverted tetrapods with different relative densities under different compressive loading conditions. Energy absorption properties under the quasistatic compression of SLMed Ti-6Al-4V 3D anti-tetrachiral auxetic lattices were investigated by Seetoh et al. [20]. The direct metal printing (DMP) process was used by Kolken et al. [21] to fabricate Ti-6Al-4V auxetic lattices based on a re-entrant hexagonal honeycomb unit cell. Structures were tested under compression to evaluate the mechanical performance.

Another recent research trend is the development of hybrid conventional-auxetic lattice structure systems. However, few studies have theoretically investigated this system [22,23,24]. In the majority of theoretical studies, only 2D analysis was considered and there has been little analytical modeling of the 3D arrays of the auxetic-conventional lattice system due to the geometrical complexity and high computational costs. Accordingly, only a few attempts have been made to investigate the mechanical performance of an AM hybrid Poisson’s ratio lattice system because the spatial tuning of PPR and NPR is still a challenging manufacturing issue. The existing research has mainly adopted 3D printed polymeric materials tested under quasi-static tension/compression. Guo et al. [23] proposed a new hybrid conventional-auxetic lattice structure system comprising of auxetic re-entrant and hexagonal components. Jin et al. [25] proposed a novel method of fabricating a polycaprolactone TE scaffold with tunable auxeticity by integrating electrospinning patterning with the fused deposition modeling process (FDM). Wanga et al. [26] proposed a novel mechanism to create a hybrid PR structure. In their work, they employed the latitude-and-longitude-inspired double-elliptic-ring structure with tunable Poisson’s ratio and Young’s modulus via tailoring the geometrical parameters. Their introduced structure was fabricated from PA12 polyamide by sintering (SLS) and tested under compression. 

In light of the 3D printed lattice structure bending studies, Ti-6Al-4V sandwich structures with regular re-entrant auxetic, octahedral, rhombic, and hexagonal lattice core geometries were processed by EBM and tested under 3-point bending by Yang et al. [27]. Horn et al. [28] studied the flexural behavior of EBMed Ti-6Al-4V rhombic lattice beams under the 4-point bending test. Rashid et al. [29] studied the flexural properties of the as-built SLMed AlSi12 uniform density lattice beams comprising circular, triangular, and hexagonal unit cells. Korshunova et al. [30] developed a CT-based numerical framework for the evaluation of the flexural behavior of the as-built SLMed 316L steel octet-truss lattice structures under 3-point bending loading. Recently, Tao et al. [31] investigated the flexural performance of the FDM-processed PLA lattice structure based on cubic diamond and cubic fluorite unit cells filled with polyurethane foam. In spite of these efforts, compared to studies of the static and dynamic compression characteristics, the flexural characteristics of lattice structures have still not been widely investigated in terms of the different unit cell types, relative densities, post-processing, and materials.

The concept of combining conventional-auxetic lattice structure systems with density grading to generate a density graded hybrid Poisson’s ratio lattice structure, producing it via LPBF, and investigating its flexural behavior is the novel and main idea of the current work. Promising applications of our proposed structure include medical implants, tissue engineering scaffolds, and cellular core sandwich panels. 

As outlined in the literature review, the majority of the mechanical testing of metallic AM lattice systems, regardless of whether regular, graded, auxetic, or hybrid, was based on compression testing because it is simple, economical, and a standard procedure for the compression testing of porous and cellular metals is available. Although the flexural properties are also very important for understanding the behavior of the lightweight structures, so far, few studies have focused on the investigation of the flexural behavior of AM auxetic lattice structures [32]. Additionally, to our best knowledge, there is a lack of studies that have explored the additive manufacturability and flexural properties of the Ti-6Al-4V lattice structures with a hybrid Poisson’s ratio. Therefore, the aim of this work was to investigate the manufacturability and flexural properties of the positive, negative, hybrid PR, and novel FG hybrid PR Ti-6Al-4V lattice structures manufactured via LPBF.

## 2. Materials and Methods

### 2.1. Material

**Figure 1:**
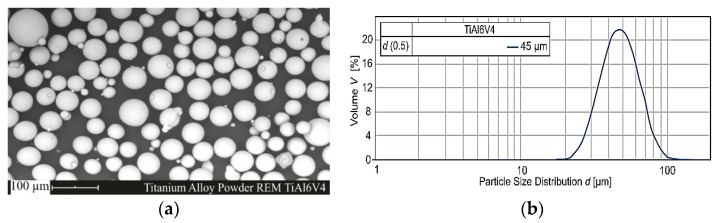
The shape and morphology of the starting powder: (**a**) SEM micrograph of the Ti6Al4V powder, and (**b**) the particle size distribution.

The material used for the individual struts and lattice structured specimens was commercial gas-atomized Ti-6Al-4V powder with a nominal particle size comprised between 10 and 45 μm. Table 1 shows the actual chemical composition as determined by means of optical emission spectroscopy at room temperature(all material data recompiled from the manufacturer (AP&C, Quebec, Canada) [33]. The scanning electron microscope (SEM) micrograph and particle size distribution by the laser diffraction of the Ti-6Al-4V powder are shown in Figure 1. As observed from Figure 1, the particles appeared spherical and smooth with a few smaller satellites attached to bigger particles.

### 2.2. Design and LPBF Manufacturing of Lattice Structures

**Figure 2:**
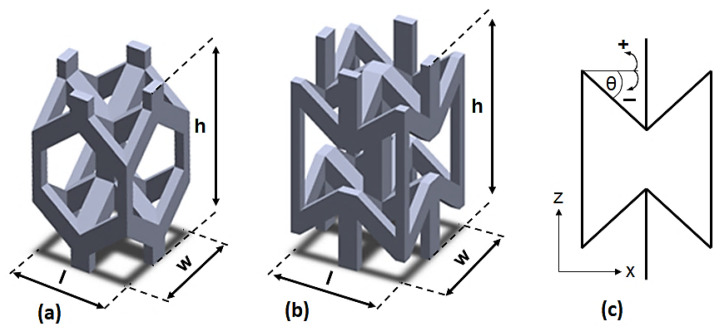
The 3D CAD models of the basic unit cells: (**a**) HU; (**b**) RU, and (**c**) the 2D representation of the re-entrant unit cell.

The CAD models of four different lattice structures were generated using Solidworks (Dassault Systèmes, Waltham, MA). Two basic unit cells were used to construct the lattice structures: (1) a conventional hexagonal unit cell (HU); and (2) a re-entrant hexagonal unit cell (RU). Figure 2a,b shows a 3D representation of the basic conventional and re-entrant hexagonal unit cells. The struts comprising the lattices were assigned with sizes corresponding to the desired relative densities and exhibited a square cross section to reduce the size of the stereolithography (.STL) files. The inclined struts of both base unit cells were aligned at angles of 40° with respect to the horizontal building platform. Figure 2c illustrates the 2D representation of the re-entrant unit cell. With reference to Figure 2c, the lattice structure is auxetic (exhibit NPR) if θ is negative (the unit cell possesses a re-entrant shape as seen in Figure 2b), and the lattice structure is conventional (exhibits PPR) if θ is positive (the unit cell possesses a hexagonal shape as seen in Figure 2a). The lattice structure is hybrid (exhibit HPR) if θ is negative in a part of the structure and positive in the remaining part. 

**Figure 3:**
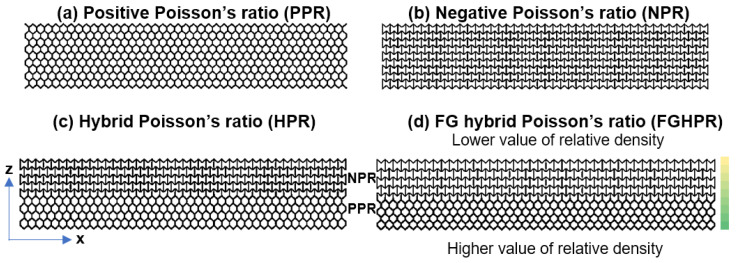
The front view of the base layer used for the creation of: (**a**) the PPR structure, (**b**) NPR structure, (**c**) HPR structure, and (**d**) FGHPR structure with a 1D graded density in the z direction.

The lattice beam configurations were designed with a similar theoretical relative density of 0.28 and bounding cube geometries of 100 mm × 20 mm × 20 mm, which were the same nominal dimensions used in the study by Shen et al. [34]. These allowed for comparisons to be made among the different samples in order to estimate the flexural properties. With reference to Figure 3, the first specimen (specimen ID: PPR) was a periodic lattice structure with a uniform density constructed of regular hexagonal unit cells, with a cell size (h × l × w) of 2.4 mm × 2.4 mm × 4.2 mm and strut size of 0.48 mm. Similarly, the second specimen (ID: NPR) was a periodic lattice structure with uniform density and consisted of re-entrant hexagonal unit cells with a cell size (h × w × l) of 2.4 mm × 2.4 mm × 4.2 mm and strut size of 0.40 mm. The third sample (ID: HPR) had a uniform density and consisted of 3D mesh combining the two basic unit cells with a cell size of 2.4 mm × 2.4 mm × 4.2 mm and strut size of 0.48 mm for HU and cell size of 2.4 mm × 2.4 mm × 4.2 mm and strut size of 0.40 mm for RU. The fourth sample (ID: FGHPR) had a 1D functionally graded density with the hybrid Poisson’s ratio. Because the structure of the FGHPR was graded in the z direction (bending loading direction), construction of the model by repetition of the base unit cells on the three directions x, y, and z is not applicable. Therefore, to generate the structural gradation in the z direction of the FGHPR lattice, a single base layer of the FGHPR structure, as shown in Figure 3d, was generated first, then patterned in the y direction with the desired distances. The same layer was placed in the xz plane then patterned in the y direction with the desired distances. The interaction between the patterned layers generated an interconnected network of the FGHBR structure. 

For the FGHBR structure, the density gradient is represented by the color code beside the front view of the FGHPR sample shown in Figure 3d. The nine layers in the xz plane were each assigned a different relative density, corresponding to a linear decrease at the base (PPR portion) toward the top (NPR portion). The overall average of these densities was 0.28. In the color code, the green color means the highest density region and yellow means the lowest density region. Because the FGHBR structure is functionally graded with a customized local relative density, generating it by means of Solidworks consumed considerable time.

For the hybrid Poisson’s ratio samples (HPR and FGHPR), the upper portion of the lattice beam in which compressive stress is dominant under 4-point bending loading consists of re-entrant unit cells, then, it will exhibit a negative Poisson’s ratio. Additionally, the lower portion that is subjected to tension consists of conventional hexagonal unit cells and will exhibit a positive Poisson’s ratio. The reason behind combining both structures in such an arrangement is that, under this arrangement, both the hexagonal and re-entrant structures will undergo transverse contraction under bending loading, which translates into significant beam thinning, which leads to a rise in beam densification and hence an increase in beam stiffness. This significant stiffening of beams is expected to cause higher plastic energy dissipation. 

In addition to the described four lattice structures, four individual inclined struts (exhibit inclination angle of θ = 40°) and vertical struts (exhibit θ = 90°) with strut sizes of 0.4, 0.42, 0.48, and 0.52 and a length of 40 mm were also designed to be manufactured via LPBF for the purpose of geometrical and hardness measurements, in addition to the microstructural characterization.

All CAD models were saved in the (.STL) format then exported to an STL editor software (Magics, Materialise, Plymouth, MI, USA) to prepare files for additive manufacturing. Subsequently, MTT AutoFab software Version 1.4 Build 7417 (Marcam Engineering Gmbh, Bremen, Germany) was used to orientate the STL CAD models before the building process, in addition to setting the specification of the laser scan strategy and the process parameters involved during LPBF. The CAD models were then merged into a single STL file and exported to the LPBF machine. The lattice and strut models were processed using an LPBF 250 HL machine (SLM Solutions GmbH, Lübeck, Germany) equipped with a 400 W yttrium fiber laser. The processing parameters used in this study are shown in Table 2. Samples were oriented on the building platform so that the build-direction during layerwise manufacturing was parallel to the 4-point bending loading axis. The chessboard scanning strategy in which the layer is divided into defined stripes was applied. As the solidified material is susceptible to containing a high level of residual stresses close to the substrate, the substrate was preheated at 200 °C. At this temperature, it is expected to relieve the thermal stresses over a few millimeters.

### 2.3. Measurements and Characterizations

***Geometrical measurements:*** After manufacturing, the samples were cleaned from adhering powder particles by compressed air and ultrasonic treatment. The bounding volume of the as-built samples was measured with a digital Vernier caliper (Mitutoyo Corporation, Kanagawa, Japan) with a 0.01 mm accuracy. The sample dimensions were derived from the average of five points on each of the as-built samples. The designed surface area of each model was retrieved from the CAD models in Solidworks. A Keyence VHX5000 Digital microscope (Keyence Deutschland GmbH; Neu-Isenburg, Germany) was used to investigate the morphologies and manufacturing quality of the lattice structures and analyze the as-built strut size. Ten dimensional values of the strut size were measured and the average value was calculated.

***Heat treatment:*** The LPBF process is characterized by short laser-powder interaction times and localized high heat input, which leads to steep thermal gradients, rapid solidification, and fast cooling (orders of thousands of degrees per second). A post-stress relief heat treatment is therefore often necessary for the as-built Ti-6Al-4V after LPBF processing to mitigate the impact of the residual stress on the mechanical property performance [35]. In this research, after the manufacturing of Ti–6Al–4V lattice beams and struts, the as-built lattice samples and some struts were subjected to a post-processing heat treatment. Before heat treatment, the as-built lattice samples were washed with isopropanol and distilled water in an ultrasonic cleaner, and then dried in air. The samples were then enclosed in custom-made vacuum quartz tubes, with a vacuum better than 10–3 mbar. Heat treatments were then executed for 2 h at 1050 °C above the β transus to allow the α and β phases to reach equilibrium, followed by furnace cooling to room temperature. The stress relief temperature and holding time were chosen according to [36] to relieve the residual stresses that occur in the LPBF parts. 

***Relative density:*** The relative density of all samples was measured experimentally according to the Archimedes principle prior to being used for mechanical evaluation by weighing each sample in air ($m_{a}$) and subsequently in isopropanol ($m_{l}$) three times independently using an electronic balance with a 0.001 g accuracy. Using the measured average masses and the known isopropanol density ($\rho_{l}$), the lattice structure volume ($V_{\mathrm{lattice}}$) can be calculated as follows:

(1) $$V_{\mathrm{lattice}}=\frac{m_{a}-m_{l}}{\rho_{l}}$$

With the lattice volume ($V_{\mathrm{lattice}}$) and the bounding volume ($V_{\mathrm{solid}}$), the relative density can be determined as follows:

(2) $$\mathrm{Relative}\;\mathrm{density}\;=\frac{V_{\mathrm{lattice}}}{V_{\mathrm{solid}}}\;$$

CAD models were also used to determine the theoretical relative density of the structures. The theoretical relative density was calculated by evaluating the ratio of the volume of the structure by that of the solid from which the cell walls were made.

***Microstructural evaluation:*** As-built and heat-treated vertical struts with a strut size of 0.52 mm were sectioned through a plane perpendicular to the building platform, and then polished down according to the standard metallographic preparation, and subsequent microstructural investigations via electron backscatter diffraction (EBSD) were then carried out. The EBSD scans were conducted using a Philips XL 40 ESEM scanning electron microscope at 20 kV on the electro polished specimens with a scanning area of 90 × 90 µm.

***Microhardness:*** Microhardness on the as-built and heat-treated vertical struts with strut sizes of 0.52 mm were measured using a Vickers tester KB 30 FA (KB Prüftechnik, Hochdorf-Assenheim, Germany) with a load of 200 g for 15 s at ten different locations for each sample to obtain an average value. 

**Figure 4:**
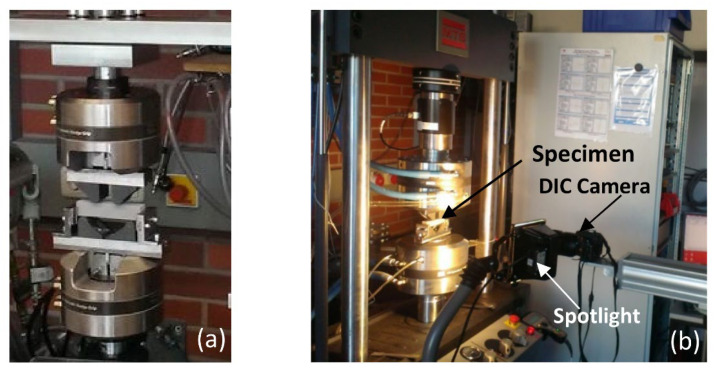
(**a**) Lattice sample mounted on the FPB test machine before the beginning of the test; (**b**) Experimental test setup accompanied by DIC.

***4-point bending (FPB):*** Four point bending quasi-static tests were conducted on the heat treated LPBFed lattice beam samples at room temperature with a crosshead displacement rate of 2 mm/min using the MTS Landmark 810 Servo hydraulic test system (MTS Systems Corporation, Eden Prairie, MN, USA), equipped with a 50 kN load cell. The two lower rollers were 70 mm spaced and the two upper rollers were 35 mm spaced and supported by a fixed base. Samples had a 100 mm length and a 20 × 20 mm^2^ cross section. During the test load and displacement, data were recorded and a digital image correlation (DIC) system was used to evaluate the displacement and strain evolution in the samples. A Nikon D60 digital camera with a resolution of 10 megapixels was adapted for the acquisition of in situ images of the sample surfaces in displacement intervals of 50 μm. Spotlight was used to ensure the proper illumination necessary for DIC analyses. Tests were terminated after ensuring that final densification occurred. Figure 4 shows the experimental test setup for the 4-point bending test with the DIC system. One sample of each configuration was tested.

After the FPB test, all images were correlated using VIC-2D DIC software (Correlated Solutions Inc., Irmo, SC, USA). The images were correlated using a subset size of 80 pixels and a step size of 10 pixels. The Tresca shear theory was used to calculate the local effective strains. 

## 3. Results and Discussion

### 3.1. Lattice Manufacturability and Morphological Characterization

**Figure 5:**
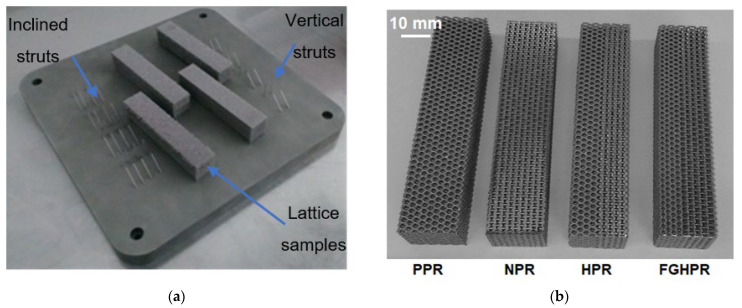
Photographs showing: (**a**) the struts and lattice samples on the LPBF building platform; (**b**) the as-built four lattice samples.

**Figure 6:**
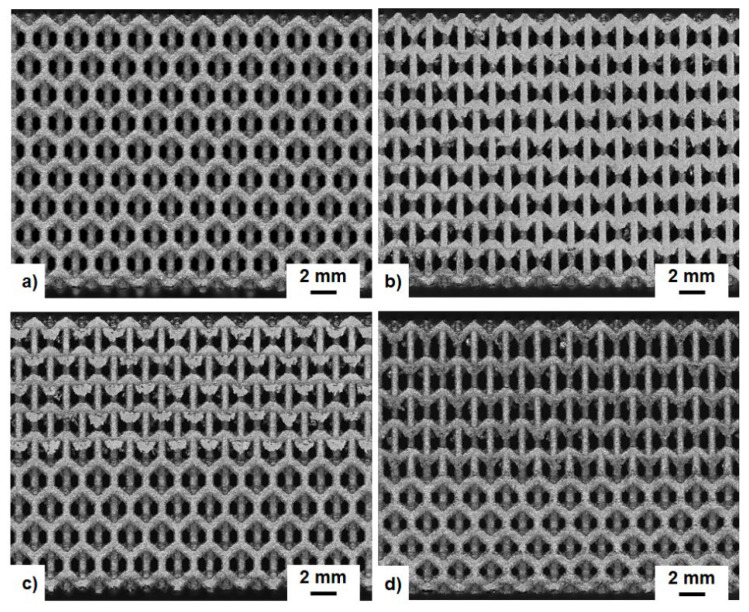
The optical microscope images of the LPBF-manufactured cellular lattice structures with the same relative density: PPR (**a**), NPR (**b**), HPR (**c**) and FGHPR (**d**). BD refers to the building direction.

Figure 5a shows the photographs of the struts and lattice samples on the 200 mm^2^ LPBF building platform directly after manufacturing, while Figure 5b shows the as-built four lattice samples after being removed from the platform. Visual inspection of the manufactured structures indicated that there were no signs of imperfections. Figure 6 shows the optical microscope images of the LPBF-manufactured lattice structures with the same relative density of 0.28. It was observed that the four types of lattice structures were successfully manufactured, proving the capability of the LPBF technique to fabricate intricate lattice structures.

**Figure 7:**
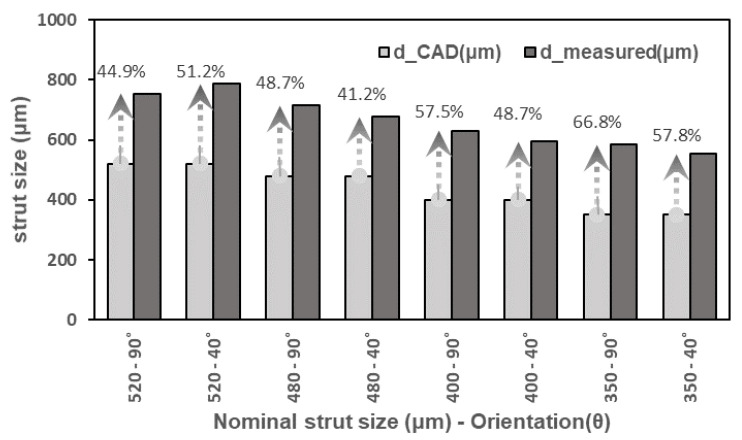
A comparison between the targeted CAD and the average of the measured strut sizes at inclination angles of 40° and 90°.

Figure 7 shows a comparison between the targeted CAD and the measured strut sizes, (d_CAD and d_measured), respectively. It was observed that both the as-built vertical and inclined struts had larger equivalent diameters than the CAD target specifications. This effect is in contrast to the EBM-processed struts [28]. It could also be observed that for the small size struts (350, 400, and 480 μm), the inclined beam thicknesses were smaller than the vertical beam thicknesses. However, for a larger size strut (520 μm), the inclined beam thickness was larger than the vertical beam thickness. The deviation from the CAD data specifications of the small thickness vertical struts were more pronounced than in the large thickness struts.

**Figure 8:**
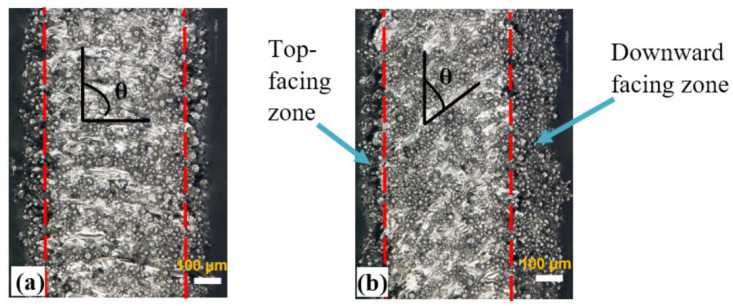
The optical microscope images of: (**a**) vertical strut (θ = 90°), and (**b**) inclined strut (θ = 40°). Zones between the red dotted lines represent the fully melted portion of the struts.

Figure 8 shows the optical microscope morphologies of the surfaces of the as-built Ti6Al4V vertical and inclined struts. Inspection of the surface roughness indicates that for vertical struts (Figure 8a), the roughness did not fluctuate significantly along the circumference of the strut. However, for the inclined struts, the roughness fluctuated significantly (Figure 8b). The downward facing surfaces of the inclined struts (surface oriented toward the build platform) exhibited a high roughness whereas the top-facing zone of the struts (surface normal oriented away from build platen) exhibited a rather low roughness (see Figure 8b). The increased roughness on the downward facing surfaces of the inclined struts was due to the energy flow into the powder bed, which led to the adhesion of a large amount of powder particles on the downward facing surfaces.

### 3.2. Relative Density and Surface Area

The relative density and surface area are key parameters controlling the properties of the lattice structures. It can be seen from Table 3 that the targeted relative densities were lower than those measured because of the increased size of the struts. Deviation between the idealized structure and the corresponding manufactured structure ranged from 17.7% for PPR to 23.4% for NPR. There was no significant difference in the measured relative density between the PPR, HPR, and FGHPR structures. A comparison of the theoretical surface area of the lattice structures investigated in this study are also shown in Table 3. The NPR sample had the highest surface area of 0.111 m^2^. The dimensional deviation between the nominal and LPBF-processed lattice designs was the main reason behind the increase in the measured relative density than the nominal one. The high surface roughness due to the adhered and partially melted powder particles and laser-induced balling phenomenon inherent to LPBF are the reasons for the dimensional deviation in LPBF [8]. Using design for AM (DfAM) approach, improving precision and reliability of AM systems, optimizing the process parameters and post-treatment are the current remedial actions to overcome this menace.

### 3.3. Microstructural Characterization

**Figure 9:**
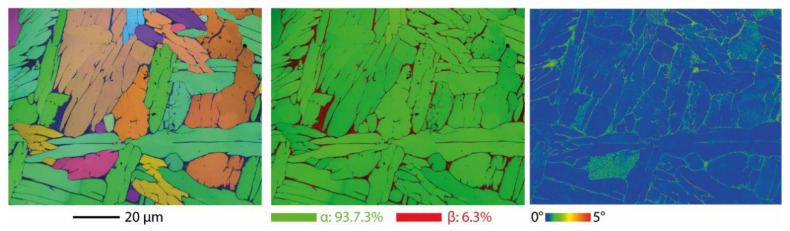
The SEM-EBSD mapping of the LPBF-processed Ti6Al4V alloy.

Figure 9 shows the EBSD images of the LPBF-processed Ti6Al4V alloy. As can be seen in the figure, the as-built Ti6Al4V exhibited the microstructure of long and narrow laths with an average length of about 20 μm. These fine martensitic laths can be interpreted as both metastable hexagonal α’ martensite and α (martensite) as the EBSD-image cannot distinguish the difference between α and α’ as they are both hexagonal. However, the low EBSD image quality indicates high internal stresses, and thus acts as an indicator for the presence of α’-titanium [35]. LPBF fabricated Ti6Al4V alloys in the as-built condition are characterized by its brittle behavior due to the limited deformation capability of hexagonal dense packed crystal structures, α and α’, which are less ductile compared with the body-centered cubic β.

The heat treatment of the struts and lattice samples were performed in vacuum at 1050 °C for 2 h and then cooled down inside the furnace. This slow cooling rate fostered the nucleation and growth of globular α grains, leading to a microstructure consisting of almost entirely coarse α-grains with fractions of β particles distributed along the grain boundaries of the α grains. An increased ductile β-phase fraction after heat treatment provided hot workability of the laser melted Ti6Al4V alloy. The average sizes of the α grain ranged from 5 μm to 25 μm and the size of the β particles was approximately 10 nm located along the grain boundaries of the α phase. This result is consistent with that reported in other studies on LPBF Ti6Al4V [35,36,37].

### 3.4. Micro-Hardness

**Figure 10:**
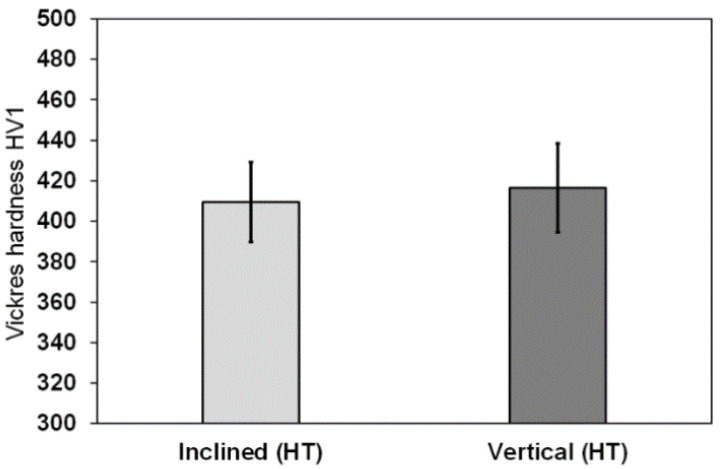
The micro-hardness for the heat-treated vertical and inclined struts.

As shown in Figure 10, the micro-hardness of the heat-treated (HT) inclined struts was 409.33 ± 19.7 HV1 and for the heat-treated vertical strut it was 416.5 ± 21.84 HV1. It is clear that the vertical struts had a slightly higher hardness than the inclined heat-treated struts, but both were greater than the hardness value reported in other investigations of the as-built Ti6Al4V struts due to the coarsening of the microstructure after heat treatment compared to the original finer α’ martensite [38]. The obtained hardness values were consistent with the hardness values reported in the other investigations [39,40]. 

### 3.5. Flexural Properties

**Figure 11:**
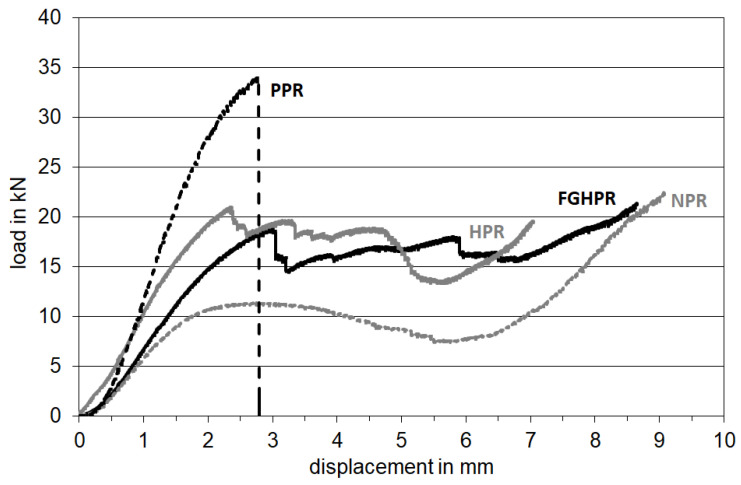
The load–displacement curves of the PPR, NPR, HPR and FGHPR lattice beam samples tested under four-point bending.

**Figure 12:**
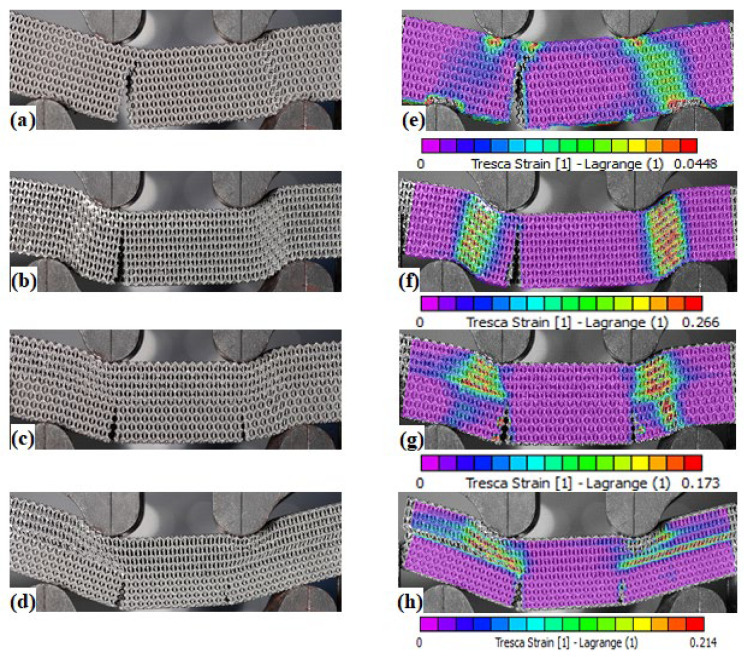
The failure modes of: PPR (**a**); NPR (**b**); HPR (**c**), and FGHPR (**d**) at the onset of final densification, and the strains measured by DIC of: PPR (**e**); NPR (**f**); HPR (**g**) and FGHPR (**h**) at the onset of final densification.

Figure 11 shows the typical load–displacement curves of the PPR, NPR, HPR, and FGHPR lattice beam samples tested under four-point bending. From the figure, it is clear that all four samples exhibited an initial linear elastic response to the applied load with higher stiffness and higher first peak load of 34 KN for the PPR sample followed by the plastic collapse (plateau) region, then final densification. Confirmed by DIC, within the elastic region, there was no damage induced on all specimens. After the linear elasticity response, the PPR sample showed a significant load drop after a very small amount of non-linear deformation due to the development of single side shear cracks at the location of the left support at a critical bending load point, where the crack propagated through the structure thickness suddenly until fracture (see Figure 12a). The PPR showed a brittle fracture failure, although it had a reasonable specific strength.

The NPR sample, however, showed an initial linear response followed by a nonlinear phase until a peak value was reached. Then, a smooth reduction in the load level without recognizable successive load dropped, truncated by a region of densification in which the stress rose steeply as the structure began to densify (see Figure 11). The failure of the NPR sample started with localized densification associated with the collapse of the cells underneath the supports, which resulted in more ductility than PPR, followed by single sided shear cracking at the left support until fracture. Figure 12b shows the failure mode of the NPR sample at the onset of final densification.

For HPR, after the first peak load, the load level maintained almost constant until descent in force occurred, followed by the final densification, which started at a displacement of about 5.6 mm. The failure of HPR started with shear cracks that developed at the locations of the left and right supports simultaneously with localized densification at the supports (see Figure 12c). Finally, for FGHPR, after the first peak load, the load level also maintained almost constant until final densification occurred. The failure of FGHPR started with the development of shear cracks at the locations of the left and right supports simultaneously with longitudinal shear flow at the transition plane between the upper auxetic portion and lower conventional hexagonal portion as a consequence of the structural non-homogeneity, in addition to localized indentation and densification at the weakest lower density auxetic layers beneath the supports. FGHPR is characterized by a long plateau region compared with all three other structures. Figure 12d shows the failure mode of FGHPR at the onset of final densification.

For both HPR and FGHPR, the presence of auxetic structure on the top portion of the lattice beam led to the localized densification and indentation, along with the axial shear at the transition between the upper auxetic and lower hexagonal portions. This enabled the crack propagation to be slowed to achieve a wider plastic-flow plateau stage and enhance the compressive resistance and energy absorption of the cellular solid.

**Figure 13:**
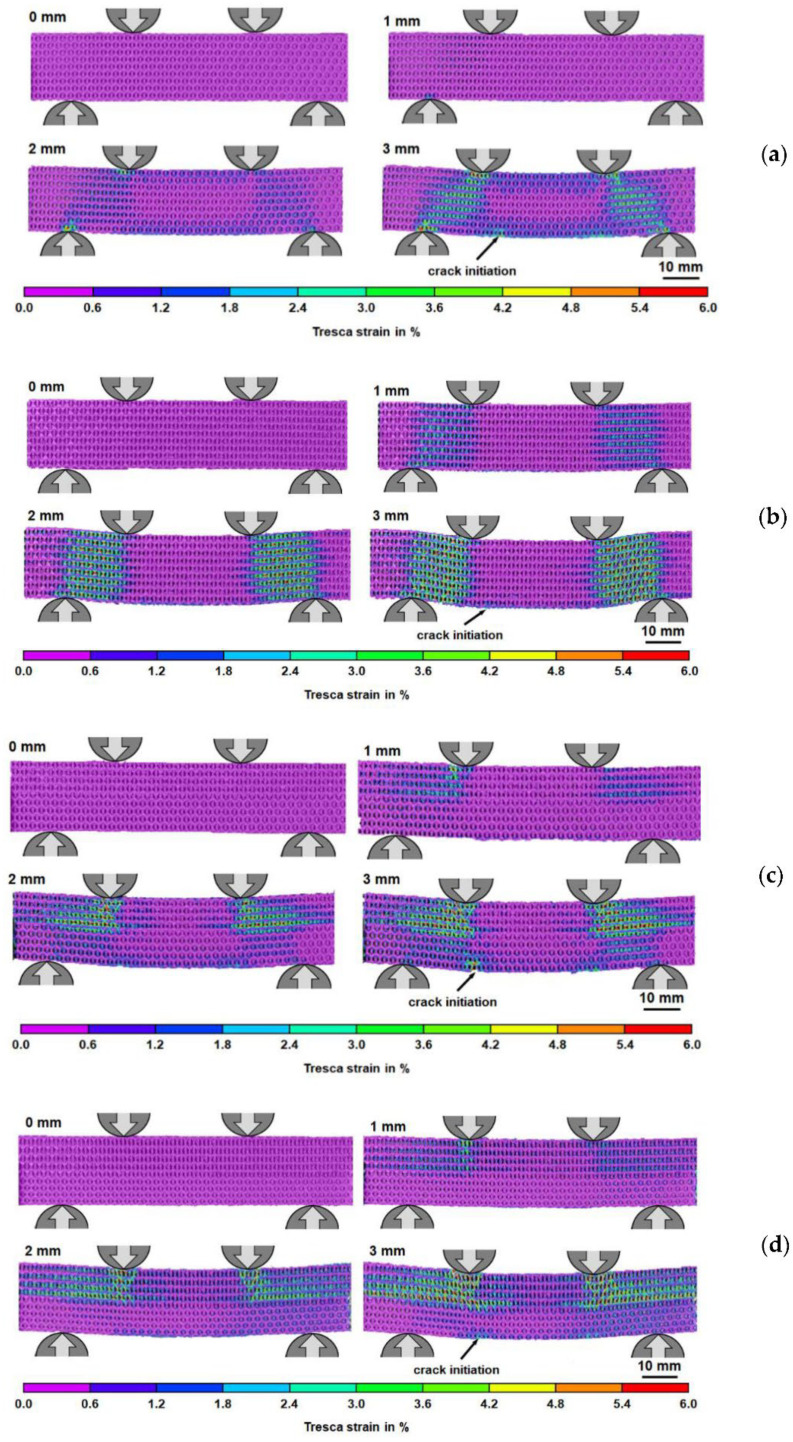
The development of the first cracks in the (**a**) PPR, (**b**) NPR, (**c**) HPR, and (**d**) FGHPR lattice structures under FPB loading.

In order to gain insights into the global and local deformation mechanisms, the failure behavior and local deformations of the lattice beams at the onset of final densification was analyzed by DIC. As shown in Figure 12e–h, the deformation and damage evolved differently in each of the four structures. Localized densification under the top supports is more pronounced for the HPR and FGHPR structures. Figure 13 shows the development of the first crack in all structures.

#### 3.5.1. Flexural Stiffness

Based on the obtained bending characteristics (maximum load in elastic range and corresponding displacement), the flexural stiffness (*EI*) was determined for each lattice structure according to the relation [41]:

$$f_{max}=\frac{F_{max\;}\times l^{3}}{8\times EI}\times \frac{a}{l}\left[1-\frac{4}{3}{\left(\frac{a}{l}\right)}^{2}\right]$$

where:

*F*_*max*_—maximum force in elastic range, N;

*f*_*max*_—The maximum elastic deflection, mm;

*l*—distance between the two outer supports, mm;

*a*—distance between the loading point and support, mm;

*E*—modulus of elasticity, MPa;

*I*—moment of inertia, mm^4^.

**Figure 14:**
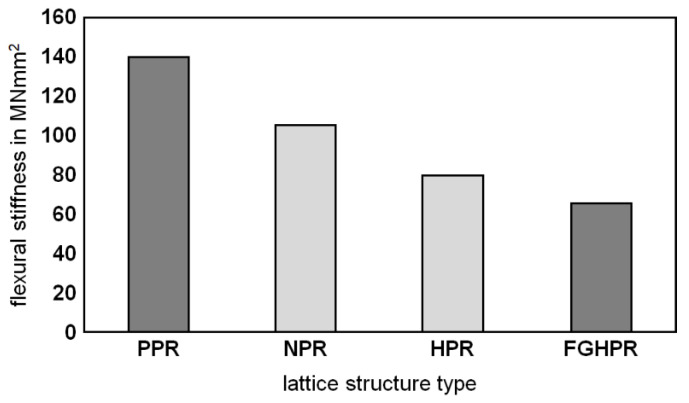
The flexural stiffness of the PPR, NPR, HPR, and FGHPR lattice structures.

Derived from this relation and the known dimensions of the structures, the flexural stiffness was calculated (Figure 14). It was observed that PPR had a higher flexural stiffness and strength than NPR, HPR, and FGHPR.

#### 3.5.2. Absorbed Energy

Lattice structures are particularly attractive for applications in the field of lightweight construction and energy absorption due to their high strength and stiffness-to-weight ratio compared to other systems. Therefore, it is important to investigate the energy absorption capability of the introduced lattice structures. In this research, the amount of energy absorption was evaluated by integrating the load deflection curves obtained by the bending tests from the start up to the limit of the onset of the final densification stages. Regarding this property, it is important for the area below the curve to be as large as possible, which means a higher load plateau region at the same time as the maximum densification strain, and this is the most attractive feature of the metal foams [41].

**Figure 15:**
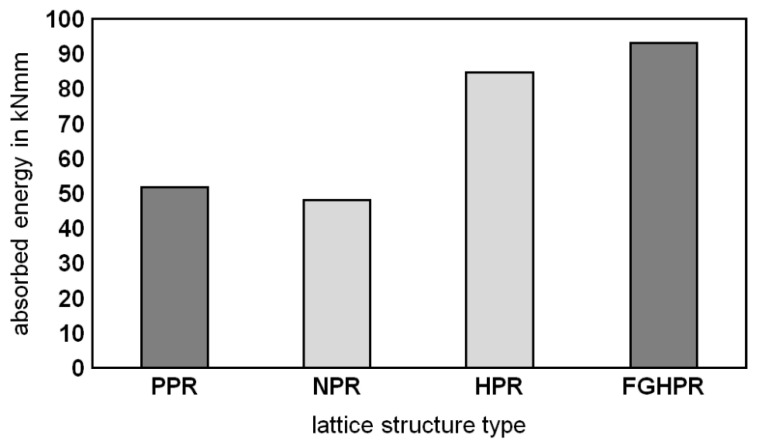
The absorbed energy of the PPR, NPR, HPR, and FGHPR lattice structures.

Figure 15 compares the energy absorbed by the four lattice samples. These results indicate that the FGHPR structure absorbed more energy than that of HPR, PPR, and NPR due to the extended stress plateau regime resulting from the mixed mode fracture condition that occurred under a combination of local indentation, shear cracking, and longitudinal shear flow (see Figure 12d). The FGHPR and HPR samples showed a 78.7% and 62.9% increase in the absorbed energy, respectively, compared to the PPR sample. A small difference in the absorbed energy was observed for PPR and NPR. 

## 4. Conclusions

In this work, four configurations of the Ti-6Al-4V lattice structures, namely PPR, NPR, HPR, and a novel functionally graded hybrid Poisson’s ratio (FGHPR) were manufactured via LPBF and investigated regarding their flexural behavior. The results were compared in terms of the flexural stiffness, absorbed energy and collapse mechanisms. The main conclusions of the present study are as follows:

1. The four types of lattice structures were successfully manufactured, proving the capability of the LPBF technique to fabricate intricate functionally graded lattices.
2. The hardness values obtained for the heat-treated LPBF-manufactured Ti-6Al-4V parts corresponded to those in the literature.
3. This study also confirmed the formation of fine acicular α/α’ of the as-built Ti6Al4V samples due to the high cooling rate in the LPBF process and evolution of the β-phase fraction after heat treatment.
4. The experimental investigation demonstrated that the PPR structure showed the highest strength.
5. The PPR structure also showed high flexural stiffness (140 MN/mm^2^), followed by the NPR, HPR, then FGHPR structures with 105 MN/mm^2^, 80 MN/mm^2^, and 65 MN/mm^2^, respectively.
6. The structural ductility of the PPR was significantly lower, which indicated that the PPR structures had a rather low energy absorption capacity. This result is consistent with the findings of Yang et al. [27].
7. Enhanced stiffness and strength of HPR and FGHPR were expected to be obtained in the case of eliminating the shear flow (slipping) between the NPR portion and PPR portion. Eliminating the shear flow may be achieved by increasing the stiffness of the intermediate layer.
8. The best response in terms of absorbed energy was obtained for the functionally graded hybrid PR (FGHPR) structure. Both the FGHPR and hybrid PR (HPR) structures showed a 78.7% and 62.9% increase in the absorbed energy, respectively, when compared to the PPR structure.
9. The experimental results also revealed that the deformation and failure mechanisms evolved differently in the four structures.

This work marks an important step forward in the understanding of the characteristics of additively manufactured metallic functionally graded hybrid Poisson’s ratio lattice structures. The results are encouraging and open many dimensions for future investigations. This research, however, is subject to the limitations that only one sample with graded density and hybrid Poisson’s ratio was investigated and compared to three uniform density samples. Therefore, future investigations will be directed toward testing the impact of different configurations of 3D porosity graded hybrid Poisson’s ratio lattice structures on the mechanical behavior and energy absorption capacity, in addition to comparing the results with a solid counterpart. 

With the continuous improvement in the accuracy of AM systems and materials, within the next few years, metallic lattice structures comprising graded density with spatial tunable PR combined with geometrical optimization is likely to become an important topic in the research of AM lightweight structures in the search for better load-carrying capacity and energy absorption.

## Figures and Tables

**Table 1:** The chemical composition of the used Ti-6Al-4V alloy.

Element	Al	V	Fe (Max.)	C	O	N	H	Ti
wt%	6.4	4	0.2	0.01	0.13	0.02	0.002	Bal.

**Table 2:** The SLM 250HL process parameters.

Laser Power (W)	Layer Thickness (μm)	Hatch Space (μm)	Energy Density (J/mm^3^)	Scan Velocity mm/s	Scan Strategy	Building Plate Temperature, °C
175	30	120	68,5	710	Chessboard	200

**Table 3:** The theoretical and measured relative density.

Lattice Type	Theoretical Relative Density	Measured Relative Density	% Difference	Theoretical Surface Area (m^2^)
PPR	0.28	0.3297	+17.7	0.094
NPR	0.28	0.3457	+23.4	0.111
HPR	0.28	0.3350	+19.6	0.103
FGHPR	0.28	0.3322	+18.6	0.102

## References

[1] Xiao D., Mu L., Zhao G. (2015). Indentation response of sandwich panels with positive gradient metallic cellular core. J. Sandw. Struct. Mater..

[2] Al-Saedi D.S.J., Masood S.H., Faizan-Ur-Rab M., Alomarah A., Ponnusamy P. (2018). Mechanical properties and energy absorption capability of functionally graded F2BCC lattice fabricated by SLM. Mater. Des..

[3] Ma X., Zhang D.Z., Zhao M., Jiang J., Luo F., Zhou H. (2022). Mechanical and energy absorption properties of functionally graded lattice structures based on minimal curved surfaces. Int. J. Adv. Manuf. Technol..

[4] Yang L., Wu S., Yan C., Chen P., Zhang L., Han C., Cai C. (2021). Fatigue properties of Ti-6Al-4V Gyroid graded lattice structures fabricated by laser powder bed fusion with lateral loading. Addit. Manuf..

[5] Chen W., Yang J., Kong H., Helou M., Zhang D., Zhao J., Jia W., Liu Q., He P., Li X. (2021). Fatigue behaviour and biocompatibility of additively manufactured bioactive tantalum graded lattice structures for load-bearing orthopaedic applications. Mater. Sci. Eng. C.

[6] Bai L., Gong C., Chen X., Zheng J., Xin L., Xiong Y., Wu X., Hu M., Li K., Sun Y. (2021). Quasi-Static compressive responses and fatigue behaviour of Ti-6Al-4 V graded lattice structures fabricated by laser powder bed fusion. Mater. Des..

[7] Choy S.Y., Sun C.-N., Sin W.J., Leong K.F., Su P.-C., Wei J., Wang P. (2021). Superior energy absorption of continuously graded microlattices by electron beam additive manufacturing. Virtual Phys. Prototyp..

[8] Mahmoud D., Al-Rubaie K.S., Elbestawi M.A. (2021). The influence of selective laser melting defects on the fatigue properties of Ti6Al4V porosity graded gyroids for bone implants. Int. J. Mech. Sci..

[9] Boldrin L., Hummel S., Scarpa F., Di Maio D., Lira C., Ruzzene M., Remillat C.D.L., Lim T.C., Rajasekaran R., Patsias S. (2016). Dynamic behaviour of auxetic gradient composite hexagonal honeycombs. Compos. Struct..

[10] Jiang W., Ren X., Wang S.L., Zhang X.G., Zhang X.Y., Luo C., Xie Y.M., Scarpa F., Alderson A., Evans K.E. (2022). Manufacturing, characteristics and applications of auxetic foams: A state-of-the-art review. Compos. Part B Eng..

[11] Rezaei S., Kadkhodapour J., Hamzehei R., Taherkhani B., Anaraki A.P., Dariushi S. (2021). Design and modeling of the 2D auxetic metamaterials with hyperelastic properties using topology optimization approach. Photonics Nanostructures-Fundam. Appl..

[12] Gao J., Xue H., Gao L., Luo Z. (2019). Topology optimization for auxetic metamaterials based on isogeometric analysis. Comput. Methods Appl. Mech. Eng..

[13] Zhang G., Khandelwal K. (2019). Computational design of finite strain auxetic metamaterials via topology optimization and nonlinear homogenization. Comput. Methods Appl. Mech. Eng..

[14] Albertini F., Dirrenberger J., Sollogoub C., Maconachie T., Leary M., Molotnikov A. (2021). Experimental and computational analysis of the mechanical properties of composite auxetic lattice structures. Addit. Manuf..

[15] Valle R., Pincheira G., Tuninetti V., Fernandez E., Uribe-Lam E. (2022). Design and Characterization of Asymmetric Cell Structure of Auxetic Material for Predictable Directional Mechanical Response. Materials.

[16] Fu M.-H., Chen Y., Hu L.-L. (2017). A novel auxetic honeycomb with enhanced in-plane stiffness and buckling strength. Compos. Struct..

[17] Warmuth F., Osmanlic F., Adler L., Lodes M.A., Körner C. (2016). Fabrication and characterisation of a fully auxetic 3D lattice structure via selective electron beam melting. Smart Mater. Struct..

[18] Shen J., Liu K., Zeng Q., Ge J., Dong Z., Liang J. (2021). Design and mechanical property studies of 3D re-entrant lattice auxetic structure. Aerosp. Sci. Technol..

[19] Novak N., Vesenjak M., Krstulović-Opara L., Ren Z. (2018). Mechanical characterisation of auxetic cellular structures built from inverted tetrapods. Compos. Struct..

[20] Seetoh I.P., Liu X., Markandan K., Zhen L., Lai C.Q. (2021). Strength and energy absorption characteristics of Ti6Al4V auxetic 3D anti-tetrachiral metamaterials. Mech. Mater..

[21] Kolken H.M.A., Lietaert K., van der Sloten T., Pouran B., Meynen A., Van Loock G., Weinans H., Scheys L., Zadpoor A.A. (2020). Mechanical performance of auxetic meta-biomaterials. J. Mech. Behav. Biomed. Mater..

[22] Lim T.C. (2002). Functionally graded beam for attaining Poisson-curving. J. Mater. Sci. Lett..

[23] Guo M.-F., Yang H., Ma L. (2020). Design and characterization of 3D AuxHex lattice structures. Int. J. Mech. Sci..

[24] Ingrole A., Hao A., Liang R. (2017). Design and modeling of auxetic and hybrid honeycomb structures for in-plane property enhancement. Mater. Des..

[25] Jin Y., Xie C., Gao Q., Zhou X., Li G., Du J., He Y. (2021). Fabrication of multi-scale and tunable auxetic scaffolds for tissue engineering. Mater. Des..

[26] Wang L., Zhu S., Wang B., Tan X., Zou Y., Chen S., Li S. (2021). Latitude-and-longitude-inspired three-dimensional auxetic metamaterials. Extrem. Mech. Lett..

[27] Yang L., Harrysson O., West H., Cormier D. (2013). A Comparison of Bending Properties for Cellular Core Sandwich Panels. Mater. Sci. Appl..

[28] Horn T.J., Harrysson O.L.A., Marcellin-Little D.J., West H.A., Lascelles B.D.X., Aman R. (2014). Flexural properties of Ti6Al4V rhombic dodecahedron open cellular structures fabricated with electron beam melting. Addit. Manuf..

[29] Rahman Rashid R.A., Mallavarapu J., Palanisamy S., Masood S.H. (2017). A comparative study of flexural properties of additively manufactured aluminium lattice structures. Mater. Today Proc..

[30] Korshunova N., Alaimo G., Hosseini S.B., Carraturo M., Reali A., Niiranen J., Auricchio F., Rank E., Kollmannsberger S. (2021). Bending behavior of octet-truss lattice structures: Modelling options, numerical characterization and experimental validation. Mater. Des..

[31] Tao Y., Li P., Zhang H., Shi S.Q., Zhang J., Yin Q. (2022). Compression and flexural properties of rigid polyurethane foam composites reinforced with 3D-printed polylactic acid lattice structures. Compos. Struct..

[32] Li C., Shen H.-S., Wang H. (2019). Nonlinear bending of sandwich beams with functionally graded negative Poisson’s ratio honeycomb core. Compos. Struct..

[33] AP&C (2020). Data Sheet: Ti-6Al-4V-MC-16-539 Powder for Additive Manufacturing.

[34] Shen Y., Cantwell W., Li Y. (2014). Skin-core adhesion in high performance sandwich structures. J. Zhejiang Univ. Sci. A.

[35] Gorny B., Niendorf T., Lackmann J., Thoene M., Troester T., Maier H.J. (2011). In situ characterization of the deformation and failure behavior of non-stochastic porous structures processed by selective laser melting. Mater. Sci. Eng. A.

[36] Brenne F., Niendorf T., Maier H.J. (2013). Additively manufactured cellular structures: Impact of microstructure and local strains on the monotonic and cyclic behavior under uniaxial and bending load. J. Mater. Process. Technol..

[37] Xu W., Lui E.W., Pateras A., Qian M., Brandt M. (2017). In situ tailoring microstructure in additively manufactured Ti-6Al-4V for superior mechanical performance. Acta Mater..

[38] Yan C., Hao L., Hussein A., Young P. (2015). Ti-6Al-4V triply periodic minimal surface structures for bone implants fabricated via selective laser melting. J. Mech. Behav. Biomed. Mater..

[39] Huang Q., Liu X., Yang X., Zhang R., Shen Z., Feng Q. (2015). Specific heat treatment of selective laser melted Ti–6Al–4V for biomedical applications. Front. Mater. Sci..

[40] Lancea C., Chicos L.A., Zaharia S.M., Pop M.A. (2017). Microstructure and micro-hardness analyses of titanium alloy Ti-6Al-4V parts manufactured by selective laser melting. Proceedings of the 4th International Conference on Computing and Solutions in Manufacturing Engineering 2016—CoSME’16.

[41] Filetin T. (2012). Bending Stiffness of Aluminium Foams. Rad Hrvat. Akad. Znan. Umjet. Teh. Znan..

